# Exploring spatial patterns of sudden cardiac arrests in the city of Toronto using Poisson kriging and Hot Spot analyses

**DOI:** 10.1371/journal.pone.0180721

**Published:** 2017-07-03

**Authors:** Raymond Przybysz, Martin Bunch

**Affiliations:** Faculty of Environmental Studies, York University, Toronto, Ontario, Canada; Azienda Ospedaliero Universitaria Careggi, ITALY

## Abstract

**Introduction:**

Our study looked at out-of-hospital sudden cardiac arrest events in the City of Toronto. These are relatively rare events, yet present a serious global clinical and public health problem. We report on the application of spatial methods and tools that, although relatively well known to geographers and natural resource scientists, need to become better known and used more frequently by health care researchers.

**Materials and methods:**

Our data came from the population-based Rescu Epistry cardiac arrest database. We limited it to the residents of the City of Toronto who experienced sudden arrest in 2010. The data was aggregated at the Dissemination Area level, and population rates were calculated. Poisson kriging was carried out on one year of data using three different spatial weights. Kriging estimates were then compared in Hot Spot analyses.

**Results:**

Spatial analysis revealed that Poisson kriging can yield reliable rates using limited data of high quality. We observed the highest rates of sudden arrests in the north and central parts of Etobicoke, western parts of North York as well as the central and southwestern parts of Scarborough while the lowest rates were found in north and eastern parts of Scarborough, downtown Toronto, and East York as well as east central parts of North York. Influence of spatial neighbours on the results did not extend past two rings of adjacent units.

**Conclusions:**

Poisson kriging has the potential to be applied to a wide range of healthcare research, particularly on rare events. This approach can be successfully combined with other spatial methods. More applied research, is needed to establish a wider acceptance for this method, especially among healthcare researchers and epidemiologists.

## Introduction

Collection of spatial data and the use of spatial methods in healthcare research have been gaining momentum in recent decades. Despite this growth, however, the number of spatial analyses that were published in major epidemiology journals in the past decade was still very low [1]. One of the reasons is that the application of spatial methods requires specific training, and this may limit their use to fields such as geography and statistics. In turn, this leads to the exclusion of such methods from healthcare research and may result in their substitution with less optimal choices when analyzing spatial phenomena. It is important, therefore, to promote spatial methods, especially those that (i) are relatively simple to use and to interpret in the field of healthcare research, (ii) have been already validated in the past, and (iii) can potentially be used in combination with other spatial tools to facilitate the interpretation of final results. These aspects were important to our study.

Our study looked at out-of-hospital sudden cardiac arrest (OHCA) events in the City of Toronto. The OHCA is a relatively rare event, yet presents a serious global clinical and public health problem. In Canada, up to 40,000 cardiac arrests occur each year [2]. In the US, the American Heart Association estimates that approximately 360, 000 people experience out-of-hospital cardiac arrests yearly that are emergency medical services–assessed [3]. However, because it’s a relatively rare event, there is a limited number of studies that looked at OHCA, especially from an ecological perspective and such research was limited mostly to mapping based on the multiple years of aggregated data [4]. Therefore, we saw an opportunity to look at OHCA events using real-world data and spatial methods such as Poisson kriging. Our intention was to derive the population rates based on one year of data only and using very small geounits, something that has not been done before not only for OHCAs but also for other rare health events.

Poisson kriging removes the instability associated with a small sample size and, thus, a high sampling variability. It has a potential, therefore, for a practical application when studying rare events or diseases. However, despite the smoothing effect of this method, the resulting maps may still be difficult to interpret, especially if a large number of geounits is involved. Additionally, Poisson kriging will not classify the areas of high risk based on statistical significance, and thus, other techniques may be required. We chose Hot Spot analyses for interpretation of our final results because this method identifies statistically significant clusters with high and low-values [5], which is an important factor in epidemiological studies. Another important factor for our selection was that this method is readily available in popular GIS software (e.g. ArcGIS) and thus, may appeal to health care researchers.

Poisson kriging was first developed by Monestiez et al. and applied in the field of marine ecology [6]. Goovaerts recognized great potential in this spatial technique and adapted it to predict cancer mortality rates in the USA [7]. He argued that this technique results in much smoother surfaces than maps of crude rates since it removes noise created by small population sizes and, therefore, minimises the artificial difference between neighbouring areas. He refined this method further in his subsequent publication when he examined cancer mortality rates among white females in Northeast USA counties [8]. One of the approaches he used was refereed to as area-to-area (ATA) Poisson kriging following a terminology presented by Kyriakidis [9]. ATA kriging takes into account the size and shape of geographical areas being analyzed and accounts for changes in variance due to population size in these areas. Because of the latter, we believe that ATA Poisson kriging can have a wide application in healthcare research which was shown in recent studies [10–12] and employed this method in our study. It is important to remember, however, that most spatial analyses, including Poisson kriging, are based on spatial associations between the studied objects, but this topic is not well discussed in the existing healthcare literature. Such research is even scarcer for Poisson kriging [13]. Therefore, the objective of our study was to enhance the knowledge of geospatial methods, tools and GIS software among health care researchers as well as to raise some important aspects of spatial analyses such as spatial weighs.

## Materials and methods

### Data

Our data came from the population-based Rescu Epistry cardiac arrest database which is comprised of data points from the Resuscitation Outcomes Consortium (ROC) Epistry-Cardiac Arrest database and the Strategies for Post Arrest Care database. The methodologies of these two databases are described elsewhere [14]. Rescu Epistry enables the comprehensive and detailed capture of all consecutive cases in a given community including the City of Toronto, and as such can be considered of high-quality. The current Rescu Epistry captures all patients with cardiac arrests or trauma for which there was a 911 response occurring in the City of Toronto and adjacent regions (Halton, Peel, Simcoe, Muskoka, York, and Durham) serving a population of 6.6 Million people. This study was approved by the Research Ethics Boards at York University and St. Michael’s Hospital in Toronto (the parent institution of Rescu).

We received a total of 6,875 records for OHCA cases attended by Emergency Medical Services (EMS) personnel between January 1 and December 31, 2010, in Toronto and the nearby regions of Durham, Muskoka, Peel, Simcoe, and York. We limited this dataset to OHCA patients who were treated by EMS and had no obvious etiology such as trauma, smoke inhalation, or industrial accidents and, thus, were considered cardiac-related (3,651 records), and then to patients who were 20 years and older (3,572 records) to align this data with Canadian census information. We considered patients’ mailing addresses as their place of residence since there was no formal residential address in the Epistry database. We geocoded these addresses using DMTI Spatial GeoPinpoint Suite 6.4 [15] and were able to code more than 98% of the OHCA records, which once again, confirmed high data quality. Lastly, we used ArcGIS 10.0 [16] and the boundary map of the City of Toronto to exclude those OHCA events that occurred outside the boundary of the City of Toronto. The resulting dataset consisted of 1,372 OHCA patients.

In the final step, we calculated the OHCA population rates in the City of Toronto using Dissemination Areas (DA), which are the smallest geographic units with available population counts (n = 3,577), to preserve as much local variation as possible. We used the Census population projections (estimated for years between the Canada census) for year 2010 that we obtained from SimplyMap [17]; the OHCA event aggregation and rate calculations were performed in ArcGIS 10.0. We had to exclude eight OHCA events from our final dataset as they were recorded in areas with no population counts. Thus, our final dataset consisted of 1,364 OHCA patients who resided in the City of Toronto at the time of their 2010 arrest in a population of 2.6 million residents, making it a rare event.

### Poisson kriging

We first ran autocorrelation analysis on the crude OHCA rates in ArcGIS 10.0 using Moran’s I and the inverse distance method to determine whether Poisson kriging was an appropriate method for our data. We chose this approach because it is one of the most common statistics to test spatial autocorrelation and is included in ArcGIS, thus making it a potentially more accessible to health researchers. We selected the inverse distance option because it is a recommended and default setting for Moran’s I calculations in most GIS and geostatistical software including ArcGIS. This method is based on a distance decay theory which means that all features influence each other but that influence decreases with distance. We chose the Euclidean measure (ordinary distance) for calculations because there are no established health care models yet, and it is the most commonly used method for continuous data [18] such as ours.

Previous studies [7] showed that Poisson kriging was a more accurate estimator when the spatial autocorrelation of the observed (crude) data was low. We then performed Area-to-Area (ATA) Poisson kriging analyses on OHCA rates using SpaceStat 3.5.6 [19]. The estimated disease rate (${\hat{r}}_{PK}$) in a given area *v*_*α*_ is calculated as a linear combination of the kernel rate *z(v*_*α*_*)* and the rates observed in *(K-1)* neighbouring areas *v*_*i*_ as:

$${\hat{r}}_{PK}\left(v_{\alpha }\right)=\overset{K}{\underset{i=1}{\sum }}\lambda_{i}\left(v_{\alpha }\right)z(v_{i})$$ (1)

where the observed disease rate is *z(v*_*α*_*) = d(v*_*α*_*)/n(v*_*α*_*)*; *d(v*_*α*_*)* is the number of observed cases; *n(v*_*α*_*)* is the size of the population at risk, and *λ*_*i*_*(v*_*α*_*)* are the weights assigned to the *K* rates. Additional details, including kriging weight calculations, can be found in Goovaerts [8] and Goovaerts and Gebreab [20]. We derived three models based on 1, 2 and 3-neighbor rings of spatial influence (first, second and third order neighbours) among all adjacent Dissemination Ares (DAs) based on spatial contiguity which is the most common method used in spatial weight calculations. The calculations are based on the number of user-specified spatial units (also called neighbor rings) that determine the extent of spatial dependence and combine the size (that determines the distance) and the shape (which determines the number of bordering neighbours) of geographical features (e.g., DAs). As these features share boundaries, they influence each other. The semivariogram and deconvolution models (not presented here) were calculated by SpaceStat software according to these three models.

An experimental semivariogram is the first step in the kriging process, including Poisson kriging. It is a mathematical formula that models the spatial trend in data. It is used to derive weights that depict the spatial autocorrelation in the existing data points to compute the interpolated values. However, there is one very important difference between Poisson and other kriging techniques: Poisson kriging is based on the aggregated data while other techniques are based on the individual data points. Thus, the point-support semivariogram must be inferred from the aggregated data via an iterative deconvolution procedure [7,20]. A detailed description and the general formulas for the deconvolution process can be found in Goovaerts and Gebreab [20].

We did not create the maps with Poisson rates because it was more important to us to detect the statistically significant clusters of high and low values of OHCA which was done in the next step.

### Hot Spot analyses

We ran the Hot Spot analyses for all three models to identify the potential locations of statistically significant areas with high and low values and to compare their locations based on 1, 2 and 3-neighbor rings of spatial influence. We carried out these analyses using the ArcGIS 10.0 tool based on the Getis-Ord Gi* statistic that is part of the software [5]. This statistic (labelled as “GiZ Score” in ArcGIS) identifies the spatial clusters (e.g., of DAs) with values higher in magnitude than one might expect to find by random chance [5].

Like Poisson kriging, Hot Spot analysis requires an assumption about the spatial relationship between the neighbouring areas. The recommended (and default) conceptualization of spatial relationships for Hot Spot analysis in ArcGIS is fixed distance [21]. We chose this method in the absence of other healthcare models. Because there is no single best way to determine the threshold (distance) for that band, we based it on the previously mentioned autocorrelation analysis that we performed for the crude OHCA rates using Moran’s I and the inverse distance method. The resulting maps were divided into seven data categories based on the Fisher-Jenks natural break algorithm that derives the optimal breaks in the data based on the lowest total error [22].

## Results

We observed more OHCA events among males than females (62.8% versus 37.2%) in our final group of patients (Table 1). The overall median age was higher among females than males (78 versus 73), and the majority (over 90%) of OHCA patients were treated in private residences (apartments, condominiums, houses and townhouses, and nursing homes). More than 40% of OHCAs were witnessed by a bystander, and 17.3% of patients had a shockable initial rhythm, (ventricular fibrillation (VF), ventricular tachycardia (VT), automated external defibrillator (AED) shock). Only 6.6% of OHCA patients survived to hospital discharge. These findings are in line with other ROC studies [23–25] except for the initial shockable rhythm which was lower in our study.

**Table 1:** Characteristics of the Toronto OHCA residents 20 years and older attended by EMS, calendar year 2010.

Characteristic	Number (percent)
**SEX**	
Female	507 (37.2)
Male	856 (62.8)
*missing*	*1*
**AGE (years)**	
Females	
median	78
range (min-max)	21–103
Males	
median	73
range (min-max)	21–101
OHCA EVENT LOCATION*	
Private residences (apartment, condominium, house, townhouse, nursing home)	1,219 (90.1)
Other (roads, public locations, offices, schools, sports venues, industrial facilities, not specified)	134 (9.9)
*missing*	*11*
**BYSTANDER WITNESSED**	
Yes	571 (41.9)
No	793 (58.1)
**INITIAL HEART RHYTHM**	
VF, VT, AED shock**	228 (17.3)
PEA, asystole, not shockable	1,092 (82.7)
*missing*	*44*
**SURVIVAL TO HOSPITAL DISCHARGE**	
Yes	90 (6.6)
No	1,269 (93.4)
*missing*	*5*
**Total number of patients**	**1,364**

AED-automated external defibrillator, PEA-pulseless electrical activity, VF-ventricular fibrillation, VT-pulseless ventricular tachycardia.

* Not always related to the place of patients’ residence.

** There were no electrocardiogram recordings from bystander-applied automatic AEDs and these were recorded as either AED shock or no shock.

There were 3,577 DAs in the City of Toronto, and less than 30% of them (996) had an OHCA event in 2010 (Table 2). The overall rate was 0.65 of OHCA events per 1,000 population with a range of 1–8 events per DA.

**Table 2:** OHCA rates per 1,000 residents 20 years and older, calendar year 2010.

OHCA rates	Number (percent)
Number of DAs with at least one OHCA event (% of all)	996 (27.8)
one OHCA event only *(% of non-missing)*	753 *(75*.*6)*
range of OHCA events (min—max)	1–8
Number of DAs without OHCA event (% of all)	2581 (72.2)
**TOTAL (n)**	**3,577**
**Overall** **OHCA** **rate (per 1,000 Toronto residents 20 years and older)**	**0.65**

**Fig 1:**
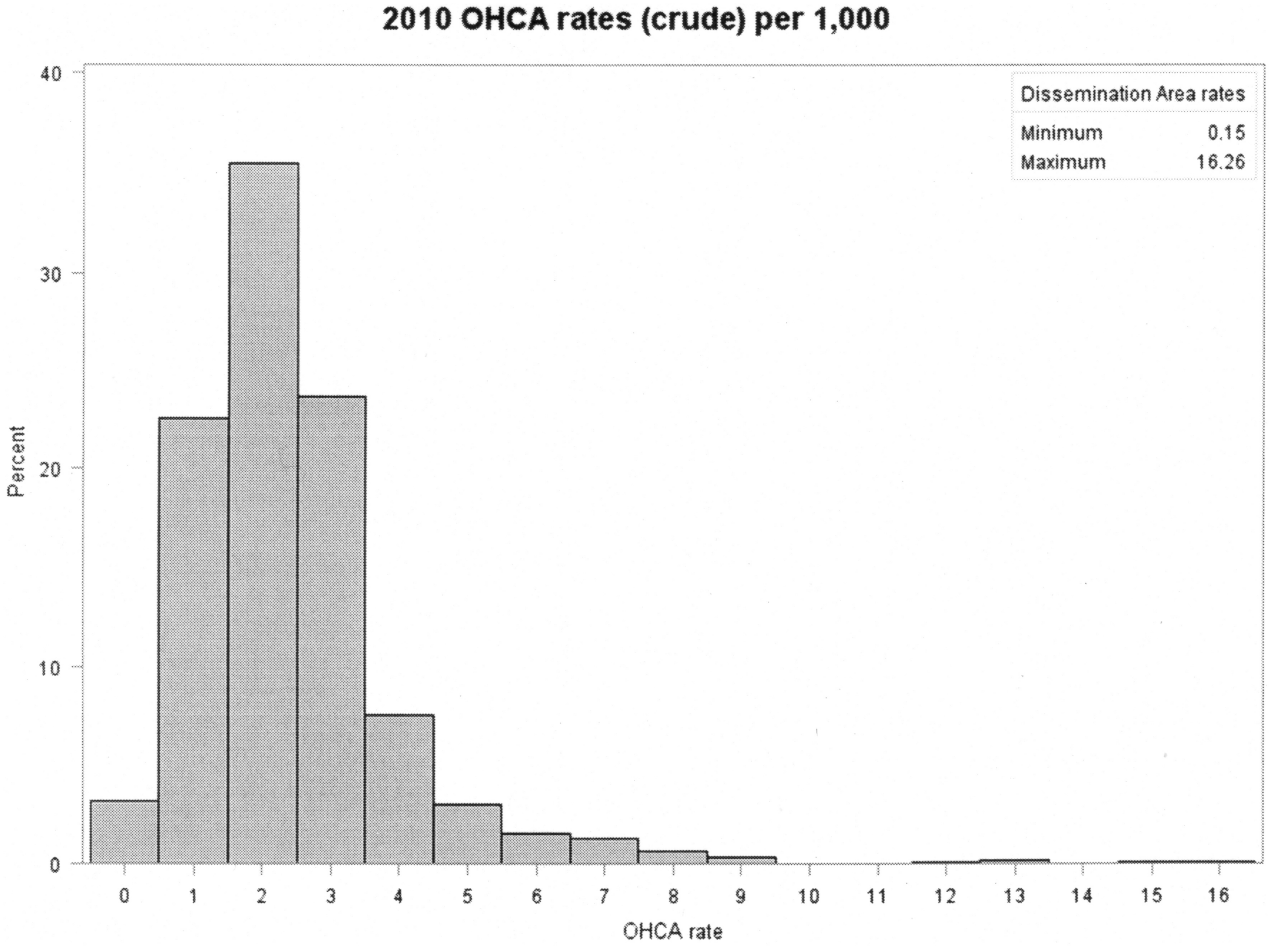
OHCA rates (crude) per 1,000 Toronto residents 20 years and older, calendar year 2010, two extreme rates (83.33 and 166.7) excluded.

Among DAs having at least one OHCA event, the crude OHCA rates ranged from 0.15 to 16.26 (with two extreme values of 83.33 and 166.7) per 1,000 Toronto residents 20 years and older (Fig 1). We decided to exclude these two extreme rates because they were based on a Census projection of just 12 residents in each of these two DAs and, as such, presented very unstable rates. However, this exclusion did not affect our Poisson kriging rates (results not presented here) because of the overall smoothing effect of this method and the fact that the unreliable rates based on small populations are smoothed more than the rates in areas with larger populations.

The crude OHCA rates showed no spatial autocorrelation (index value = 0.00, p = 0.19), indicating that Poisson kriging was a suitable method for our data. The p-value was not statistically significant, possibly meaning that the OHCA rates were not randomly distributed across the City of Toronto in 2010. This, however, was caused by the two extreme values of 83.33 and 166.7; when we removed these values, the Moran’s I was 0.02 and p<0.01.

The Poisson kriging analyses resulted in 3,188 (89.1%) DAs with non-null OHCA rates for the 1-neighbor ring analysis (Table 3). As the area of spatial influence increased to 2 and 3-neighbor rings, there were substantially fewer DAs with null OHCA rates; 3,536 (98.9%) and 3,541 (99.0%), respectively. The range of OHCA rates was quite similar for all three models, with the smallest range for 2-neighbor rings of spatial association. We also observed some negatives rates from our Poisson models ranging from 1 to 35 DAs, the highest number coming from the three-ring analysis (Table 3). The negative estimates may occur in kriging analyses due to negative weights or highly structured histograms that ignore inherent randomness (nugget effect). Some authors [26] suggested that such estimates are highly undesirable and should be assigned a zero value. We followed this advice and set all negative OHCA rates to zero for Hot Spot analyses. The resulting Poisson kriging OHCA rates ranged from 0.00 to 4.97 for the one-ring association, from 0.00 to 4.55 for two-ring and from 0.00 to 4.76 for three-ring weights (Table 3). The range of kriging variance (estimation error) was quite similar for 1, 2 and 3-neighbor ring models and ranged from 0.03 to 1.25 among DAs (results not presented).

**Table 3:** Poisson kriging rates per 1,000 Toronto residents aged 20 years and older, calendar year 2010; 1-, 2-, and 3-ring neighbor spatial associations.

Spatial weights	Number (percent)
**1-ring**	
Number of DAs with non-zero OHCA rate (% of all)	3,188 (89.1)
negative OHCA rate set to zero (% of all)	1 (<0.1)
range of positive OHCA rates (min—max)	0.00–4.97
Number of DAs with null OHCA rate (% of all)	389 (10.9)
**2-ring**	
Number of DAs with non-zero OHCA rate (% of all)	3,536 (98.9)
negative OHCA rate set to zero (% of all)	29 (0.8)
range of positive OHCA rates (min—max)	0.00–4.55
Number of DAs with null OHCA rate (% of all)	41 (1.1)
**3-ring**	
Number of DAs with non-zero OHCA rate (% of all)	3,541 (99.0)
negative OHCA rate set to zero (% of all)	35 (1.0)
range of positive OHCA rates (min—max)	0.00–4.76
Number of DAs with null OHCA rate (% of all)	36 (1.0)
**TOTAL (n)**	**3,577**
Number of DAs without residents (%)	10 (0.3)
Number of DAs with residents (%)	3,567 (99.7)
population 20 years and older (min–max)/DA	2–12,558
**Overall** **OHCA** **rate (per 1,000 Toronto residents 20 years and older)**	**0.65**

**Fig 2:**
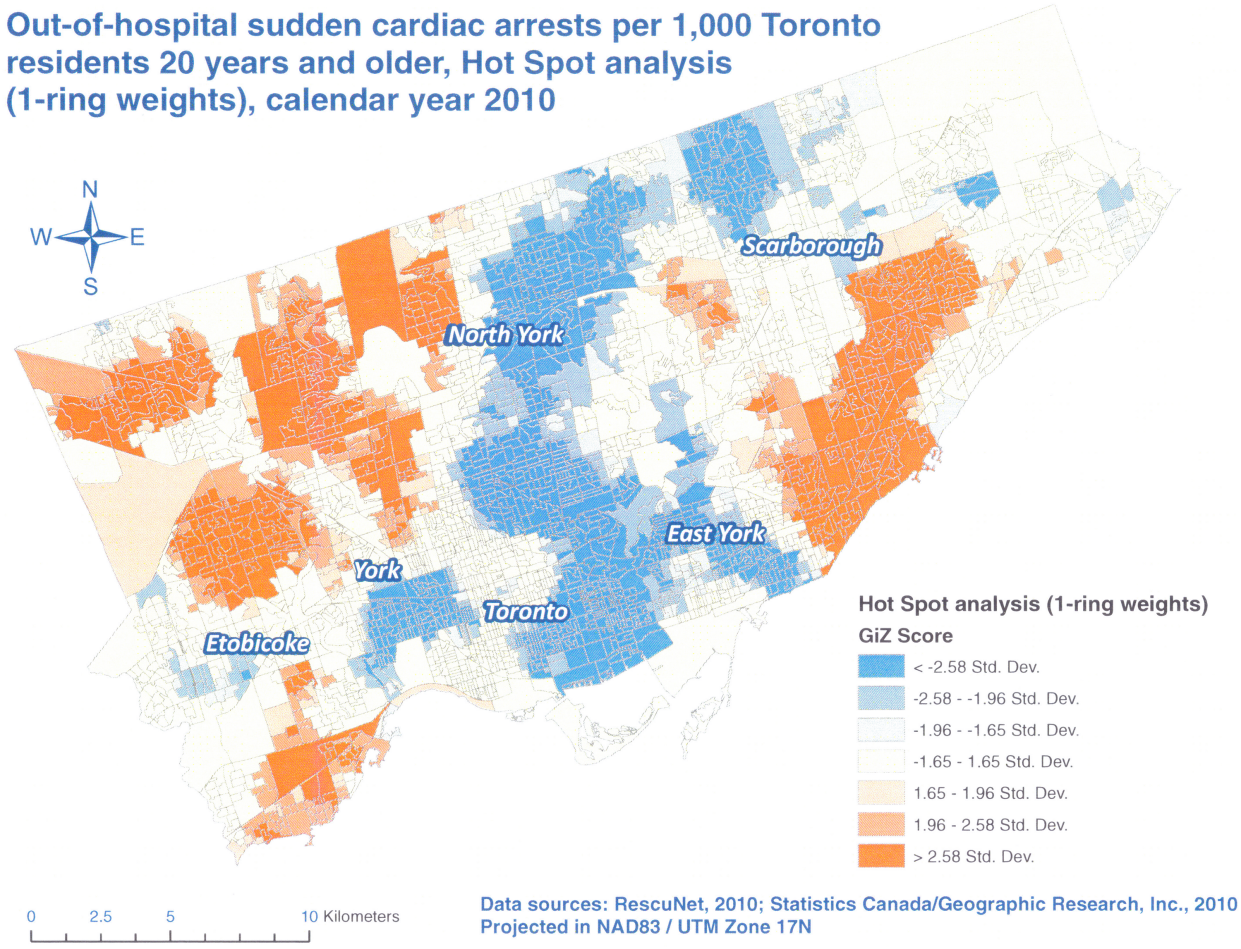
Hot Spot analysis, Poisson kriging, 1-neighbor ring spatial weights, OHCA rates per 1,000 Toronto residents 20 years and older, calendar year 2010.

**Fig 3:**
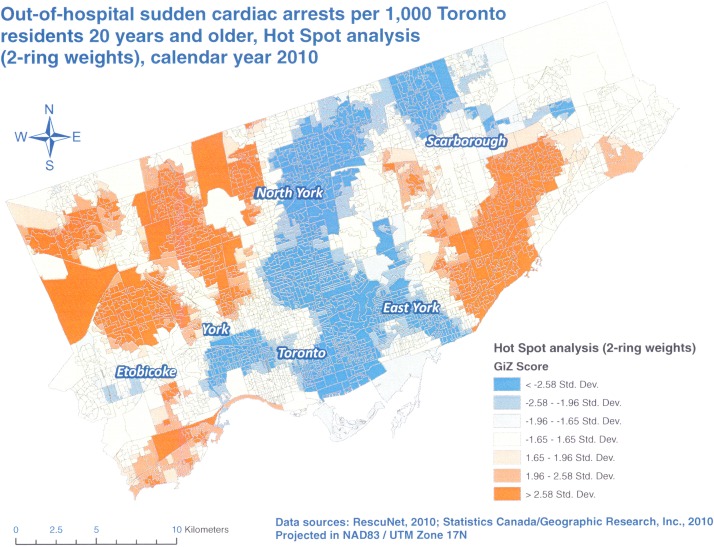
Hot Spot analysis, Poisson kriging, 2-neighbor ring spatial weights, OHCA rates per 1,000 Toronto residents 20 years and older, calendar year 2010.

**Fig 4:**
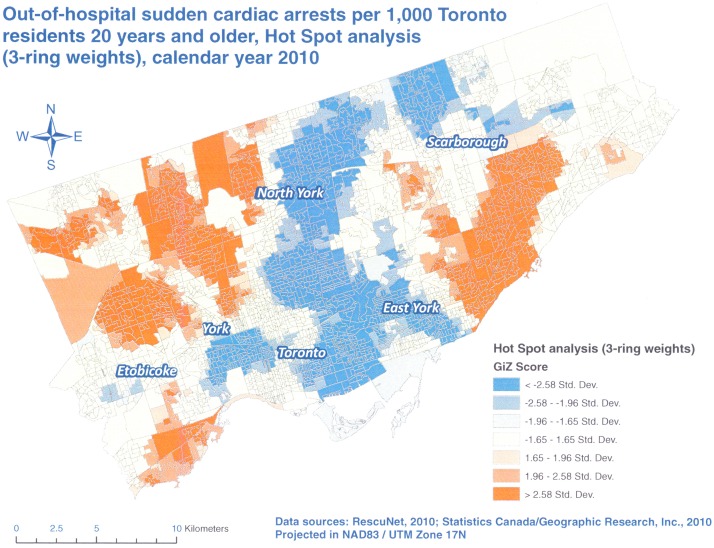
Hot Spot analysis, Poisson kriging, 3-neighbor ring spatial weights, OHCA rates per 1,000 Toronto residents 20 years and older, calendar year 2010.

Our Hot Spot analyses identified several areas of the City of Toronto with higher (red) and lower (blue) than expected OHCA rates in 2010 (Figs 2 to 4). All three analyses resulted in the identification of the same areas of concern, and as anticipated, the results for 2 and 3-neighbor ring models were more pronounced than those for 1-neighbor weights. However, there was no substantial difference between 2 and 3-neighbor ring models. This finding was in line with a study by Hampton and others [13] who showed that spatial dependence did not extend beyond two spatial units in their research.

We discovered that the highest rates of OHCAs were concentrated in the north and central parts of Etobicoke, western parts of North York as well as the central and southwestern parts of Scarborough (Figs 2 to 4). The lowest rates of OHCAs were found in north and eastern parts of Scarborough, downtown Toronto, and East York as well as east central parts of North York. We suspect that these differences are due to the mix of various factors in these areas such as residents’ age, percentage of male residents, presence of nursing homes, education, income and others. Since we have access to such information at the DA level, we plan further analyses (e.g. using Geographically Weighted Regression) to test these assumptions.

## Discussion

Our final results revealed similar spatial patterns of sudden cardiac arrests in the City of Toronto, to those discovered by the team of researchers from St. Michael’s Hospital in Toronto [4]. Similar to our study, the authors used the Epistry database and looked at patients 20 years and older who had experienced sudden cardiac arrest in Toronto. The major difference between these two studies was that Allan et al. [4] based their analyses on five years of aggregated data to show the average rates in large neighbourhoods, while our study used Poisson kriging to derive such rates based on one year of data only and very small geounits (DAs). However, both studies identified almost identical spatial patterns of high and low-risk areas of sudden cardiac arrests, thus, confirming that Poisson kriging can potentially yield reliable results for rare events using limited data of high quality. Additionally, it provides an opportunity to preserve personal information while maintaining patients’ privacy (i.e. hiding the actual address used to determine the appropriate DA). More than 75 percent of DAs with non-missing values in our study had only one OHCA event and thus, even after aggregation, offered a patient-level information such as sex or age while protecting their privacy. This presents a great opportunity for future studies, especially those with a small number of patients or events.

Despite this potential advantage, there are also some limitations to the spatial methods that we used in our analyses. The most important limitation of all spatial interpolators, including Poisson kriging, is a potential loss of fine detail as described by Goovaerts in many of his publications [8,27–29]. It is important, therefore, to select an appropriate scale (size) of the geographical unit being studied. Another important fact to consider is that the semivariogram in Poisson kriging is based on the areal rates rather than on the point data and that its experimental semivariogram is based on the deconvolution process of this areal data. Thus, it is impossible to know what the difference would be if a point-based semivariogram was used, unless point data exists [30]. Thus, it might be impossible to determine such difference when looking at specific events or diseases from a population perspective.

In our study, we explored three different weights. We observed more negative rates with larger areas of spatial influence of 2 and 3-neighbor rings but at the same time, these two models resulted in less null rates. Some authors suggest that the negative rates from kriging can be avoided if a more realistic semivariogram is used [26]. However, in the case of Poisson kriging this is not possible because its semivariogram is based on the deconvolution process of areal (aggregated) rates and not on the individual points. Thus, this is an inherent problem with Poisson kriging. Based on our results however, we believe that the 2- neighbor rings provided the most optimal balance between the number of negative OHCA rates that we had to set to zero and the numbers of DAs with null rates (Table 2) as well as the granularity and clarity of the results (Fig 3). By comparison, 1- neighbor ring weights resulted in a very high number of null rates, which may be true for a single year of data but this is not likely if these rates were averaged over several years which would be a simple form of rate smoothing. On the other hand, 3- neighbor rings provided similar results to two-rings, meaning that the area of spatial influence did not extend beyond 2 rings in our study.

We believe that the concept of spatial weights needs further investigation so as to understand the influence of spatial neighbours at different sizes of geounits especially with respect to healthcare research. For example, it is important to explore whether different geounits would lead to similar results. It seems that the spatial dependence in Poisson kriging models in our study did not extend beyond the two rings of neighbours, but this may be because of a large number of spatial units in our research. Additionally, we chose some default settings in ArcGIS 10.0 (e.g. inverse distance). Even though they are the most commonly used and recommended methods for continuous data such as ours, there are no well-established health care models yet so it is very difficult to determine whether these were the best choices for modelling of OHCAs. Thus, we cannot conclude that we would have seen similar results if have made different choices or used larger geounits in our study. Also, the usefulness of Poisson kriging maps can be limited when a large number of geounits is involved as such maps might be too granular for visual inspection even after the smoothing. Furthermore, the Poisson kriging maps offer only a visual interpretation of results and thus, other methods such as Hot Spot analyses need to be used when statistical significance is important.

We employed Hot Spot analyses, to identify the areas with significantly higher and lower risk of sudden cardiac. While this can be considered as another step in validation of final results, it is also important to remember that Hot Spot analysis is a smoothing tool in itself. Its application on the already smoothed Poisson kriging rates may result in over-smoothing and therefore, further reduce the local variation. Additionally, Hot Spot analyses cannot determine the factors that are associated with high-risk clusters and therefore, regression analyses might need to be employed to explore these factors.

## Conclusions

Our analyses showed that Poisson kriging, especially when combined with other spatial tools such as Hot Spot analyses might be an important and useful method to advance ecological knowledge on rare events, especially in situations where collection of a multiple-year data is not feasible. We join several authors who have demonstrated the efficacy of Poisson kriging in their studies [10–12]. We conclude that (i) Poisson kriging has the potential to be applied to a wide range of healthcare research, particularly with respect to rare events, (ii) more applied research such as ours is needed to establish a wider acceptance for this method, especially among healthcare researchers and epidemiologists, and (iii) could be successfully combined with other spatial methods. Also, our study gives us a further opportunity to investigate the effects of personal-level characteristics (e.g. covariates presented in Table 1) as well as some additional risk factors at the DA level on OHCA rates in the City of Toronto using other special methods (e.g. Geographically Weighted Regression).
